# The relationship between methane emission and daytime-dependent fecal archaeol concentration in lactating dairy cows fed two different diets

**DOI:** 10.5194/aab-63-211-2020

**Published:** 2020-07-02

**Authors:** Lisa-Marie Sandberg, Georg Thaller, Solvig Görs, Björn Kuhla, Cornelia C. Metges, Nina Krattenmacher

**Affiliations:** 1 Institute of Animal Breeding and Husbandry, Kiel University, Kiel, 24098, Germany; 2 Institute of Nutritional Physiology, Leibniz Institute for Farm Animal Biology (FBN), Dummerstorf, 18196, Germany; 3 Chair of Nutritional Physiology and Animal Nutrition, Faculty of Agricultural and Environmental Sciences, University of Rostock, Rostock, 18059, Germany

## Abstract

Archaeol is a cell membrane lipid of methanogenic archaea excreted in feces and is therefore a potential biomarker for individual methane emission (MEM). The aims of this study were to examine the potential of the fecal archaeol concentration (fArch) to be a proxy for MEM prediction in cows fed different diets and determine if the time of fecal collection affected the archaeol concentration. Thus, we investigated (i) the variation of the fArch concentration in spot samples of feces taken thrice within 8 h during respiration chamber measurements and (ii) the effect of two diets differing in nutrient composition and net energy content on the relationship between fArch and MEM in lactating cows. Two consecutive respiration trials with four primiparous and six multiparous lactating Holstein cows were performed. In the first trial (T1) at 100±3 d in milk (IM), a diet moderate in starch and fat content was fed for ad libitum intake, whereas in the second trial (T2) at 135±3 d IM, cows received a diet lower in starch and fat. Individual MEM (g d${}^{-1}$) was measured for 24 h. Fecal samples were taken at 06:30, 10:00, and 14:30 LT and analyzed for fArch using Soxhlet lipid extraction and GC–MS. Cows produced less methane (364 g ${\mathrm{CH}}_{4}$ d${}^{-1}$) during T1 and had significantly lower fArch concentrations (37.1 µg g${}^{-1}$ dry matter; DM) compared to T2 (392 g ${\mathrm{CH}}_{4}$ d${}^{-1}$ and 47.6 µg g${}^{-1}$ DM). A significant positive relationship between fArch (µg g${}^{-1}$ fecal DM) and MEM, expressed on a dry matter intake (DMI) basis (g kg${}^{-1}$ DMI), was found ($R^{2}=0.53$, n=20). Among samples collected over the day, those collected at 10:00 LT provided the best coefficient of determination for MEM ($R^{2}=0.23$). In conclusion, fArch offers some potential in serving as a proxy for innovative breeding schemes to lower enteric methane when fecal samples are taken at a certain time of the day, but more data on the sources of variation of the MEM : fArch ratios are required.

## 1 Introduction

Methane from the enteric fermentation of livestock contributes to approximately 17 % of the global methane emissions from natural and anthropogenic sources (Knapp et al., 2014). It was suggested that genetic selection of cattle has the potential to reduce methane emissions by 9 % to 19 % (Knapp et al., 2014; Pickering et al., 2015). Breeding for lower methane output requires an accurate quantification of methane emission in thousands of animals to generate a reference population. Respiration chambers are considered the gold standard for methane measurements because of their high accuracy and the ability to investigate aspects of feeding and nutrition (Storm et al., 2012). However, respiration chambers are only available in a limited number of research institutions and have a limited capacity, which restricts their applicability for methane phenotyping on a large number of individuals. Thus, cost-effective, simple, and precise phenotyping methods, applicable under farm conditions, are required (De Haas et al., 2011; Oddy et al., 2014). Preferably, a noninvasive proxy should represent a complex process such as methanogenesis in the rumen as much as possible. Microorganisms of the domain archaea are responsible for the enteric methane emission (MEM). Their cell membrane component, archaeol, is detectable in feces. Gill et al. (2010, 2011) have been the first to suggest that fecal archaeol concentration (fArch) could be used as a proxy for MEM in ruminants. In the studies of McCartney et al. (2013a and b), a positive relationship between fArch and MEM could be observed, but the relationship was weak within diet. Schwarm et al. (2015) also found only a weak relationship but concluded that fArch has a certain potential for predicting the MEM of individual animals. It has been shown that dry matter intake (DMI) is the main factor explaining MEM in ruminants (Knapp et al., 2014), and, because DMI determines water intake, the latter should show a relationship with MEM. Thus, it seems logical that DMI and water intake are also associated with fArch. However, considering all published data, Negussie et al. (2017) came to the conclusion that the fArch approach is unlikely to be useful in assessing MEM at the individual level. In contrast, we argue that the knowledge of the relationship between archaeol concentration and MEM in lactating cows is still limited and the practical potential of fArch for breeding purposes has not yet been sufficiently studied. In the previous studies, total feces collections were made over several days for fArch analysis (Gill et al., 2011; McCartney et al., 2013b; Schwarm et al., 2015). However, total feces collections are not practical under farm conditions. Thus the objectives of our explorative study were (i) to analyze spot samples of feces and explore if fecal archaeol concentrations change during the course of a day and (ii) to examine the effect of two diets differing in nutrient composition known to divergently affect methane production in dairy cows in established lactation on the relationship between fArch and MEM.

## 2 Material and methods

### 2.1 Experimental design and feeding

Two consecutive feeding trials were performed on 10 lactating German Holstein dairy cows in first (n=4) or second (n=6) established lactation. The experimental protocol was in accordance with the German Animal Welfare Act guidelines for the use of animals as experimental subjects and was approved by the State Government in Mecklenburg-Western Pomerania (Registration No. LALLF M-V/TSD/7221.3-1.1-034/12).

Since calving and during the first trial (T1) at 100±3 d in milk (IM), cows were fed a total mixed ration (TMR) for ad libitum intake containing 178 g starch per kilogram dry matter (DM) and 44 g crude fat per kilogram DM. From 105±3 d IM onwards, the diet was changed, and the same cows received a TMR lower in starch and fat content (99 g starch per kilogram DM, 27 g crude fat per kilogram DM). After a mean adaptation time of 35 d, the second trial (T2) at 135±3 d IM was performed. The ingredients and chemical composition of the corn-silage-based and grass-silage-based diets were described in detail by Aguinaga Casañas et al. (2015). Milk yield was recorded at each milking; milk aliquots of the afternoon and morning milkings were pooled for the analysis of milk composition (percent of milk fat, protein, and lactose) measured by LKV, Güstrow, Mecklenburg-Western Pomerania. The energy-corrected milk yield (ECM) was calculated as ((0.038× grams of fat + 0.024× grams of protein + 0.017× grams of lactose) × kilograms of milk)/3.14 (Reist et al., 2002).

In T1 MEM was measured at 100±3 d IM, whereas in T2 cows were at 135±3 d IM when MEM was measured for 24 h each in open-circuit respiration chambers at the Leibniz Institute for Farm Animal Biology (FBN) (Derno et al., 2009). This was part of a larger study on the time course of MEM, the results of which will be published separately. Cows were halter-trained and acclimatized several times to the chambers before the first measurement. The ambient temperature, hydrostatic pressure, and relative humidity in the chambers were kept at 15±1 ${}^{\circ }$C, 1010±15 hPa, and 60 %, respectively. Light was on between 06:00 and 19:00 LT. Feed was provided at 07:00 and 15:00 LT. Cows were milked twice daily at 06:30 and 16:30 LT and had free access to water. The day before the start of MEM measurements, the body weight of cows was determined immediately before being placed in the chambers at 14:30 LT. On the next (1st) day, daily feed intake, water intake, and MEM data were recorded, and MEM measurement started at 15:00 LT and continued until 15:00 LT of the following (2nd) day. Prior to and between the respiration trials, the animals were housed in a freestall barn. The methane concentration was measured every 6 min by infrared absorption, and MEM (in L d${}^{-1}$) was calculated (Derno et al., 2013). We converted the MEM unit liter to gram using the average methane density of 0.7 g L${}^{-1}$. Methane emission phenotypes were also expressed as methane yield (MY; g ${\mathrm{CH}}_{4}$ kg${}^{-1}$ DMI) and methane intensity (MI; g kg${}^{-1}$ ECM). The DMI and ECM values were recorded within the same 24 h measurement period as MEM was measured. The information on milk composition was missing for one cow in T2. During the 2nd day of MEM measurement in the respiration chamber, fecal spot samples were collected at three times (06:30, 10:00, and 14:30 LT) by rectal grabbing (approx. 200 g) and immediately frozen at -20 ${}^{\circ }$C. The reason for the timing was the assumption that archaea responsible for MEM at a certain point in time will be defecated at a later time when they have passed the gastrointestinal tract. Prior to archaeol analysis, the stored fecal samples were thawed, dried for 72 h at 60 ${}^{\circ }$C, and ground to pass a 1 mm screen. The quantification of fArch was performed as described by Görs et al. (2016) and was expressed as micrograms per gram (µg g${}^{-1}$) fecal DM.

### 2.2 Statistical analysis

Data were analyzed using the REG and GLM procedures of SAS (Version 9.4, SAS Institute Inc., Cary, NC) (SAS Institute Inc., 2013). First, fixed effects of lactation number and trial were tested separately by ANOVA. The effect of the trial represents the combined effects of diet type and stage of lactation because no crossover design was used. As the effect of the trial was significant for several traits, appropriate least squares means were estimated and tested for the difference. Other authors argued that a (extended) lactation number could affect the level of MEM (e.g., Garnsworthy et al., 2012), but we could not detect differences either in MEM or fArch between cows in first and second lactation. ANOVA was also applied to water intake and DMI for the traits MEM and fArch. Next, linear regression analysis was applied to consider the relationship between 24 h MEM and average fArch, calculated from three spot samples obtained on the 2nd day at 06:30, 10:00, and 14:30 LT. To investigate whether the time of the day the fecal spot sample was collected had an effect on the relationship with MEM, fArch from different sampling times were analyzed separately with regression analysis. The following model was used to estimate simultaneously the significant effect of the trial and fArch:

1 $$y_{ij}=\mu +{\mathrm{trial}}_{i}+b\times x_{ij}+e_{ij},$$

where $y_{ij}$ is the dependent variable MY or MI, trial${}_{i}$ is the fixed effect of T1 or T2, which were coded as 1 and 2, respectively, b is the regression coefficient for dependent variables on fArch, $x_{ij}$ is the covariate of fArch of individual j within trial i, and $e_{ij}$ is the random residual effect. Statistical significance of the model was accepted at P<0.05.

## 3 Results and discussion

A summary of the fArch concentration data (mean fArch and fArch of the three individual samples) is given in Table 1. The mean fArch was 42.4 µg g${}^{-1}$ fecal DM (SD = 8.2 µg g${}^{-1}$ fecal DM), ranging from 28.1 to 61.7 µg g${}^{-1}$ fecal DM, which was remarkably higher than reported by Gill et al. (2011) (concentrate-based 5.1 and grass-silage-based 30.6 µg g${}^{-1}$ fecal DM), McCartney et al. (2013b) (mean 9.2 µg g${}^{-1}$ fecal DM), and Schwarm et al. (2015) (mean 16.8 µg g${}^{-1}$ fecal DM). Different levels of fArch are probably due to differences in DMI, the concentrate to forage ratio, performance levels, and life stages of the animals, as well as the archaeol analysis methodology. In the study of McCartney et al. (2013b), Holstein-Friesian and Jersey × Holstein heifers in early and mid-lactation with considerably lower milk yield and DMI than in the present study were investigated. The DMI in the studies of Gill et al. (2011; 9.2 and 11.4 kg) and Schwarm et al. (2015; 9.2 and 9.9 kg) was lower than in our study (T1: 16.3 kg; T2: 14.8 kg). Görs et al. (2016) have shown that sample extraction using the Soxhlet procedure, compared to a sonication-aided extraction, was twice as efficient and might explain partly the higher fArch determined in the present study compared to the other studies (McCartney et al., 2013b; Gill et al., 2011; Schwarm et al. 2015). It should be noted that the development of a standardized analytical procedure is crucial especially for the pooling and comparison of fArch data from different research sites. Internationally agreed guidelines are a prerequisite for using fArch as a MEM proxy for breeding purposes.

**Table 1:** Descriptive statistics for the fecal archaeol concentration.

Trait${}^{*}$	Mean	SD	Min.	Max.
Average fArch, µg g${}^{-1}$ fecal DM	42.4	8.2	28.1	61.7
06:30 LT fArch, µg g${}^{-1}$ fecal DM	42.3	8.6	23.5	61.3
10:00 LT fArch, µg g${}^{-1}$ fecal DM	41.3	11.4	26.2	77.2
14:30 LT fArch, µg g${}^{-1}$ fecal DM	47.1	10.2	35.1	69.1

${}^{*}$ fArch = fecal archaeol concentration; 06:30 LT fArch = fArch for the fecal sample taken at 06:30 LT; 10:00 LT fArch = fArch for the fecal sample taken at 10:00 LT; 14:30 LT fArch = fArch for the fecal sample taken at 14:30 LT.

In agreement with the findings of Gill et al. (2011) and Schwarm et al. (2015), the diet with the moderate starch and higher fat levels (T1) resulted in significantly lower fArch levels (P=0.002) accompanied by a lower, albeit not statistically significant, MEM and a lower MY (P=0.001; Table 2). The trial number also affected milk yield and MI (P<0.05; Table 2) in part reflective of the slight difference in DMI between T1 and T2. It has been shown previously that diets high in starch and fat reduce MEM in cows (Johnson and Johnson, 1995; Van Gastelen et al., 2015; Benchaar et al., 2015). Diets with high starch and fat contents decrease ${\mathrm{CH}}_{4}$ output via a lower hydrogen production available for methanogens and decrease rumen pH, which can inhibit the growth of methanogens and ciliate protozoa (Knapp et al., 2014). Furthermore, a greater dietary fat content might also be associated with decreases in fiber degradability and the toxic effects of unsaturated fatty acids on archaea and with a reduction in metabolic activity of archaea and thus ${\mathrm{CH}}_{4}$ production (Maia et al., 2007; Benchaar et al., 2015).

**Table 2:** Least squares means with standard error of difference (SED) for trial effects on cow performance, fecal archaeol, methane production, and efficiency traits.

Trait${}^{1}$	Trial${}^{2}$		SED	P-value
	T1	T2		
Dry matter intake, kg	16.3	14.8	1.43	0.312
Water intake, L d${}^{-1}$	65.9	62.5	5.07	0.511
Milk yield, kg	30.8	25.0	2.26	0.019
ECM, kg	34.4	27.6	2.35	0.011
Milk fat, %	4.69	4.66	0.31	0.948
Milk protein, %	3.40	3.07	0.08	0.010
MEM, ${\mathrm{CH}}_{4}$ g d${}^{-1}$	364	392	31.7	0.402
MY, g ${\mathrm{CH}}_{4}$ kg${}^{-1}$ DMI	22.5	26.6	1.08	0.001
MI, g ${\mathrm{CH}}_{4}$ kg${}^{-1}$ ECM	10.7	14.8	1.26	0.005
Average fArch, µg g${}^{-1}$ fecal DM	37.1	47.6	2.85	0.002
06:30 LT fArch, µg g${}^{-1}$ fecal DM	38.3	46.3	3.50	0.035
10:00 LT fArch, µg g${}^{-1}$ fecal DM	36.7	45.8	4.77	0.071
14:30 LT fArch, µg g${}^{-1}$ fecal DM	39.4	54.8	2.98	0.000

${}^{1}$ ECM = energy-corrected milk yield; MEM = methane emission; MY = methane yield; MI = methane intensity; fArch = fecal archaeol concentration, 06:30 LT fArch = fArch for the fecal sample taken at 06:30 LT; 10:00 LT fArch = fArch for the fecal sample taken at 10:00 LT; 14:30 LT fArch = fArch for the fecal sample taken at 14:30 LT. ${}^{2}$ Two consecutive respiration trials with 10 cows, where T1 = respiration chamber measurement at 100±3 d in milk (IM) with a diet moderate in starch and fat content and T2 = respiration chamber measurement at 135±3 d IM with a diet lower in starch and fat content.

To date only a few reports on fArch as a proxy for methane emission in cattle are available. Simple linear regression analysis for MEM and the average fArch of three spot samples showed a weak positive relationship with $R^{2}=0.16$ (P=0.07, n=20), as similarly found by Schwarm et al. (2015) ($R^{2}=0.19$, P=0.08, n=14). Water intake affected MEM positively (P<0.001), which is not surprising because water intake and DMI are highly correlated traits (Kramer et al., 2008), but fArch was independent of water intake (P=0.842). The lack of a strong relationship between fArch and MEM may result from variations in the methane-producing activity of methanogens (Aguinaga Casañas et al., 2015) along the total gastrointestinal tract and the appearance of methanogens in the feces, which in turn is affected by DMI, passage rate of digesta, nutrient intake, and nutrient-specific digestion kinetics. Furthermore, in addition to the membrane lipid archaeol, representatives of the newly discovered methanogenic Methanomassiliicoccus group possess butane- or pentanetriol dibiphytanyl tetraether lipids, which we could not analyze with our GC–MS method (Becker et al., 2016). Because these methanogens were present in ruminants (Henderson et al., 2015; Kelly et al., 2016) albeit with unclear physiology and quantitative importance among methanogens, missing the contribution of Methanomassiliicoccales to methanogen lipid concentrations may potentially explain some of the variation between MEM and fArch. Moreover, this variation might also be a consequence of fecal sampling. In various studies sampling was either done as quantitative collection over 6 d (McCartney et al., 2013b), pooling of single grab samples over several days (Gill et al., 2011), or pooling of several 24 h fecal samples over a week (Schwarm et al., 2015). In a study by McCartney et al. (2014), the fecal samples from the grazing cow's dung were collected in the pasture over 5 d and pooled. In contrast, we collected and analyzed three samples grabbed at different times of the day (06:30, 10:00, and 14:30 LT) when MEM was measured simultaneously. Pooling of fecal excretions for 24 h or feces collection over several days is not feasible under farm conditions and also because farmers would be unlikely to adopt practices requiring great effort and without production benefits (Hristov et al., 2013). In routine testing of individual animals, the use of fArch derived from an adequate spot sample would be most suitable. Görs et al. (2016) proposed that the level of fArch content could be influenced by the time interval between the last meal and the time of the fecal collection. The more feed a cow consumes in a relatively short period of time, the higher the passage rate through the rumen-intestinal tract will be (Jentsch et al., 2007), thereby reducing the contact time of methanogens with feed particles and other microbiota in the rumen and resulting in less MEM per unit feed ingested. It has been shown that MEM is immediately stimulated by a feed intake event but also by feed composition and intake long before actual methane measurements (Kuhla et al., 2015). When considering only one single spot sample from the day in the regression analysis, the highest coefficient of determination was obtained when fecal samples were taken at 10:00 LT ($R^{2}=0.23$, P=0.03), while those of the other days were substantially lower and not significant ($R^{2}=0.0$5 at 06:30 LT, $R^{2}=0.02$ at 14:30 LT). In view of our findings that the closest relationship between 24 h MEM and fArch occurred with the fecal sample taken at 10:00 LT, the time of sampling is of importance. It should be noted that the 10:00 LT fecal sampling was the one closest in time to the provision of feed (07:00 and 15:00 LT) and that the time interval between feeding and sampling might be crucial in this context.

At a comparable level of DMI, diets with a higher proportion of concentrate are more rapidly fermentable, which results in a higher digesta passage rate, in a shorter contact time between feed particles and methanogens, and thus in lower MEM and fArch (Colucci et al., 1990; Gill et al., 2011; Goopy et al., 2014). Moreover, more rapidly fermentable carbohydrates with a more rapid postprandial decrease in ruminal pH also lower the number of cellulolytic bacteria, resulting in less fiber degradation, proportionally less acetate and more propionate (thus also less free hydrogen), and, finally, less methane because propionate serves as a hydrogen sink. McCartney et al. (2013b) suggested that a high MEM to fArch ratio in ruminants which are fed diets with a high concentrate proportion is indicative of a selective retention of methanogens in the rumen. In the present study, we made a similar observation as in the study of Gill et al. (2011), where steers, having been fed a ration with concentrate for ad libitum intake, emitted much less methane on a DMI basis and had a lower fArch than steers fed a ration with grass silage ad libitum. This was reflected when accounting for the trials as a fixed effect in the model for the analysis of the relationship between MY (g ${\mathrm{CH}}_{4}$ kg${}^{-1}$ DMI) and fArch, which resulted in a significant positive relationship and explained 53 % of the variation in MY (P=0.001, n=20) with the following equation:

2 $$\begin{array}{rl} \mathrm{MY}\left(g\;{\mathrm{kg}}^{-1}\;\mathrm{DMI}\right) & =14.44(\mathrm{SE}=2.76,P=0.00)\\ & +0.15(\mathrm{SE}=0.08,P=0.10)\times \mathrm{fArch}\\ & +2.59(\mathrm{SE}=1.35,P=0.07)\times \mathrm{trial}. \end{array}$$

**Figure 1:**
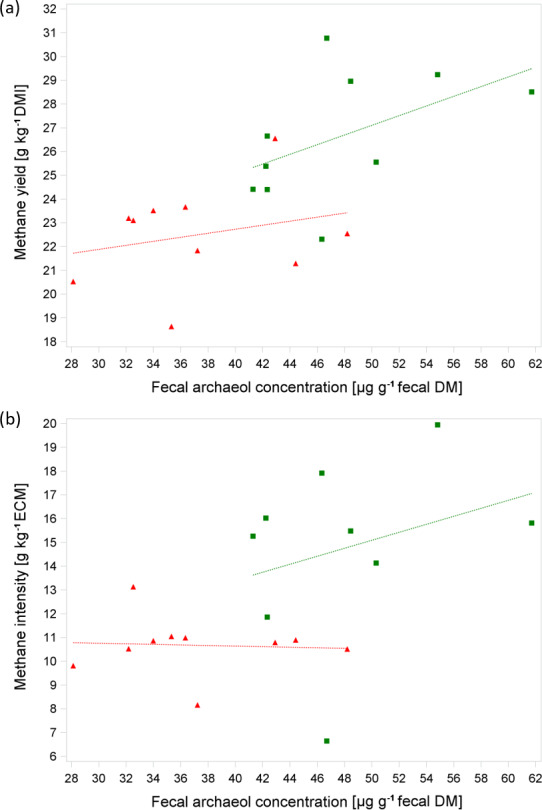
**(a, b)** Relationship between methane yield (in g kg${}^{-1}$ DMI) or methane intensity (in g kg${}^{-1}$) energy-corrected milk yield (ECM) and average fecal archaeol concentration from all three samples per cow (in µg g${}^{-1}$ fecal DM) based on 10 cows in two consecutive respiration chamber trials (trial 1: red triangle, diet with moderate starch and fat content at 100±3 d IM; trial 2: green box, diet with low starch and fat content at 135±3 d IM). Regression lines were not significantly different.

Figure 1a shows the linear relationship between MY and fArch with separate regression lines for the trials. The moderate coefficient of determination of $R^{2}=0.53$ is in the same range as that observed by Gill et al. (2011) ($R^{2}=0.55$) and as reported in the study of McCartney et al. (2013b) ($R^{2}=0.56$), irrespective of the archaeol analysis method used. The level of feed intake is a well-known determining factor for MEM (e.g., Kriss, 1930) and, as discussed above, also affects fArch. Furthermore, the level of milk performance plays an important role with regard to the phenotypical expression of both the MEM and fArch as milk yield is moderately to highly correlated with DMI in mid-lactation (Krattenmacher et al., 2019; Li et al., 2018). For breeding purposes, methane production relative to milk yield is of special interest. When animals' productivity is improved through nutrition, management, reproduction, or genetics, the methane emission per unit of product is reduced (Boadi et al., 2004). Thus, with an improvement of productivity, emission intensity decreases (Martin et al., 2010; Moate et al., 2015). This is in-line with the results of our study, showing that MI is significantly lower during T1 which is characterized by higher milk yields compared to T2 (30.8 kg vs. 25.0 kg; Table 2 and Fig. 1b). The relationship of MI and fArch was derived by the following equation ($R^{2}=0.40$, P=0.016, n=19):

3 $$\begin{array}{rl} \mathrm{MI}\left(g\;{\mathrm{kg}}^{-1}\;\mathrm{ECM}\right) & =4.50(\mathrm{SE}=3.38,P=0.20)\\ & +0.08(\mathrm{SE}=0.11,P=0.46)\times \mathrm{fArch}\\ & +3.25(\mathrm{SE}=1.73,P=0.08)\times \mathrm{trial}. \end{array}$$

Surprisingly, with increasing ECM due to higher feed intake and accordingly with a higher starch and fat intake, we observed a dilution effect for fArch in T1; i.e., the analysis of MI and fArch showed a slightly negative relationship. However, it should be noted that this relationship was not significant.

A reduction of methanogenesis or rather methanogens in the rumen should be associated with a reduction of fArch. It has been shown in some but not all studies that the abundance of methanogens in the rumen, either assessed as mcrA (methyl-coenzyme M reductase subunit α) mRNA or as the copy numbers of the 16S rRNA gene in DNA, is positively correlated with the MY and that the dietary concentrate level is inversely associated with archaeal abundance (McCartney et al., 2013a; Wallace et al., 2014; Aguinaga Casañas et al., 2015). In turn, it can be assumed that fArch and the abundance of methanogens should be related, but this relationship is also subject to variation. It was found that feces had higher concentrations of archaeol than rumen fluid fractions, or solid and liquid-associated ruminal microbes, and there was no significant relationship between fecal and ruminal archaeol concentrations (McCartney et al., 2014; Görs et al., 2016). Görs et al. (2016) speculated that this difference is due to the accumulation of archaea resident not just in the rumen but also in the small and large intestine, and McCartney et al. (2014) assumed that an under-sampling of archaea from the rumen is the reason for this discrepancy. It is therefore suggested that fecal archaeol samples do reflect the methanogen abundance of the total gastrointestinal tract better than that of the rumen only. However, it should be noted that the total number of microorganisms in the small intestine, compared with the rumen, is very low, and in the large intestine, although total bacterial numbers are very high, there is relatively little methane production. Data from sheep fed a forage-only ration indicate that only 13 % of total methane is produced in the large intestine (Murray et al., 1976).

## 4 Conclusions

Across diets, the relationship between fArch and MEM was indicative of the potential for archaeol to be a proxy for individual methane emission. Fecal archaeol represents an associated trait to enteric methanogenesis, but it cannot be implemented easily on a farm because diet composition, DMI, and the time for fecal sampling have to be taken into account. More research is needed, including a larger variation in dietary treatments and more observations with a higher number of animals repeatedly sampled. The effect of the stage of lactation should be investigated. Further, to improve our knowledge about the variation, robustness, and repeatability of archaeol, the relationship with feed intake, milk production, and other traits relevant to breeding needs to be studied.

## Data Availability

No data from a third party were used. The original data of the paper are available upon request from the corresponding author.
